# Data quantity governance for machine learning in materials science

**DOI:** 10.1093/nsr/nwad125

**Published:** 2023-05-01

**Authors:** Yue Liu, Zhengwei Yang, Xinxin Zou, Shuchang Ma, Dahui Liu, Maxim Avdeev, Siqi Shi

**Affiliations:** School of Computer Engineering and Science, Shanghai University, Shanghai200444, China; Shanghai Engineering Research Center of Intelligent Computing System, Shanghai200444, China; School of Computer Engineering and Science, Shanghai University, Shanghai200444, China; School of Computer Engineering and Science, Shanghai University, Shanghai200444, China; School of Computer Engineering and Science, Shanghai University, Shanghai200444, China; School of Computer Engineering and Science, Shanghai University, Shanghai200444, China; Australian Nuclear Science and Technology Organisation, Sydney 2232, Australia; School of Chemistry, The University of Sydney, Sydney 2006, Australia; State Key Laboratory of Advanced Special Steel, School of Materials Science and Engineering, Shanghai University, Shanghai200444, China; Materials Genome Institute, Shanghai University, Shanghai200444, China

**Keywords:** machine learning, data governance, data quantity, materials science

## Abstract

Data-driven machine learning (ML) is widely employed in the analysis of materials structure–activity relationships, performance optimization and materials design due to its superior ability to reveal latent data patterns and make accurate prediction. However, because of the laborious process of materials data acquisition, ML models encounter the issue of the mismatch between a high dimension of feature space and a small sample size (for traditional ML models) or the mismatch between model parameters and sample size (for deep-learning models), usually resulting in terrible performance. Here, we review the efforts for tackling this issue via feature reduction, sample augmentation and specific ML approaches, and show that the balance between the number of samples and features or model parameters should attract great attention during data quantity governance. Following this, we propose a synergistic data quantity governance flow with the incorporation of materials domain knowledge. After summarizing the approaches to incorporating materials domain knowledge into the process of ML, we provide examples of incorporating domain knowledge into governance schemes to demonstrate the advantages of the approach and applications. The work paves the way for obtaining the required high-quality data to accelerate materials design and discovery based on ML.

## Teaser

This paper proposed a synergistic materials governance flow with incorporation of domain knowledge to provide a high-quality data foundation for accelerating materials design and discovery.

## INTRODUCTION

Data-driven machine learning (ML) is widely employed in development of novel materials due to its ability to quickly, accurately and cheaply reveal ‘composition–structure–process–property’ relationships [1]. Note that its performance depends heavily on the quality and quantity of the input data (i.e. samples). Because obtaining material samples relies on tedious experiments or labor-intensive acquisition, the sample size is commonly small. On the other hand, materials experts usually select multiple descriptors (i.e. features) to study complex structure–activity relationships, resulting in a high dimension of the feature space of samples. Hence, keeping the balance between the number of features and the sample size is vital for the discovery and design of novel materials using data-driven ML. Note that the above balance mainly concentrates on the research employing traditional ML models because this type of model has limited ability for data analysis and is profoundly affected by the number of features and the size of the samples. When it occurs in deep-learning (DL) models, thanks to their excellent ability for information extraction, the major concern will transfer to the balance between the sample size and the model scales (i.e. the number of model parameters).

Herein, we summarize 107 papers in materials science, containing 109 data sets, to illustrate the statistics on the data-set size, as shown in Supplementary Fig. S1. The size of ∼57% of the data sets is <500, ∼67% of data sets comprise <1000 samples and only ∼21% of data sets contain >2000 samples. Next, we analyse the ratio of the number of features to the sample size. In ∼35.8% of the data sets, the ratio exceeds 1/4 and for ∼8% of the data sets it exceeds >1. This indicates that in most cases, even though the sample size is four times larger than the number of features, the issue of ‘high dimensionality of feature space vs. small data set size’ is obviously encountered in ML applications for materials science. Meanwhile, as the model scale increases, the demand for training data will grow exponentially. Hence, the balance between the sample size and the number of model parameters should also be valued, for which the solutions fall into data augmentation, learning algorithm modification and the improvement of data representation patterns.

To alleviate the problem of the poor ratios of the feature space dimensionality to the data-set size, researchers usually focus on eliminating redundant features, e.g. feature selection [2] and dimensionality reduction [3]. This facilitates keeping a proper balance between the number of features and samples so that ML models can easily mine latent patterns in the context of small samples [4]. However, these methods mainly rely on statistical approaches, namely they cannot directly assess which features are most relevant. For example, the single-variable correlation analysis method can only identify features that are highly correlated with each other. However, it cannot determine which features should be retained due to the coupling of multiple features of the structure–activity relationships. Therefore, incorporating materials domain knowledge into the methods becomes necessary for the guidance of feature selection.

In addition, focusing on governing the number of samples can also be an effective approach to keeping a proper balance with the number of features or model parameters. This can be categorized into sample-oriented and model-oriented methods. The sample-oriented methods aim to augment or select samples to facilitate ML modeling, e.g. generative adversarial network (GAN) [5], auto-encoder (AE) [6] and active learning [7]. The model-oriented methods aim to change the learning patterns of the model that can capture the latent information in a few-shot context, e.g. ensemble learning [8], transfer learning (TL) [9].

Though the research related to data quantity governance is beginning to be conducted, there is still a lack of governance flow to systematically and comprehensively improve the volume of data in materials science. This study aims to introduce and review common data quantity governance methods for materials science dealing with the balance between data-set size and feature space dimensionality (or the number of model parameters), including feature reduction, sample augmentation and specific ML approaches. We also show that incorporating materials domain knowledge into the learning process allows the construction of ML models with explainability, robustness and generalization, and illustrates corresponding incorporating approaches. Following this, we propose a synergistic data quantity governance flow with the incorporation of materials domain knowledge, aiming to govern the data quantity in an accurate and explainable way. The examples are provided on the integration of domain knowledge into the data-driven ML model to further improve reliability and prediction accuracy.

## DATA QUANTITY GOVERNANCE METHODS IN MATERIALS SCIENCE

**Figure 1.**
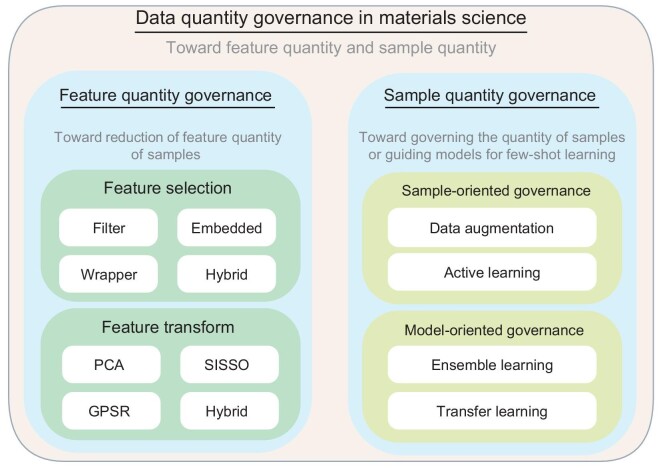
The main aspects of data quantity governance.

In this section, the data quantity governance methods employed in materials science are detailed (shown in Fig. 1), separately focusing on sample and feature quantity governance.

### Feature quantity governance

Feature definition is one of the foundations for ML modeling. However, defining a large number of features is not guaranteed to improve the performance of ML models. That is, in high-dimension feature space, redundant features with high correlation may produce a negative impact on the prediction performance and explainability of the ML model. Therefore, it is a critical issue to effectively govern features to reduce the feature quantity (i.e. the number of dimensions of feature space) while at the same time enabling the model to mine general patterns latent in the materials data. The common approaches focus on feature selection (FS) and feature transform (FT).

#### FS

FS is a critical preprocessing step to identify and rank the most relevant features, and can be generally categorized into filter, embedded, wrapper and hybrid methods. Here, we illustrate examples of the research on FS in materials science in Table 1.

**Table 1.** Examples of feature selection.

Types	Methods	Materials	Number of raw features	Number of governed features	Number of samples	Results	Reference
Filter	PCC	Pb-free perovskites	32	20	540	RMSE: 0.322	[10]
	PCC and SCC	Cubic perovskite oxides	5	3	1376	*R* ^2^: 0.87	[11]
	IE	Steels	25	–	437	*R* ^2^: 0.98	[12]
	MIC	High-temperature alloys	466	21	166	Accuracy: >90%	[13]
Embedded	L1-Norm	Carbon fiber	6	4	500	RMSE/AV: 2.55%	[16]
	GBR	Nickel-based single-crystal superalloys	21	14	94	*R* ^2^: 0.90	[17]
	RF	High-temperature superconductors	145	5	∼16 400	Accuracy: ∼90%	[18]
	LASSO	Single-atom catalyst	333 932	75	91	RMSE: 0.4096	[19]
	FeaLect	FCC and HCP	34	10	–	AUC: 80.75	[20]
Wrapper	ANN	FCC solute	111	14	218	RMSE: 0.092	[22]
	RF	Crystalline materials	452	187	952	*R* ^2^: 0.92	[23]
	Cluster	Equiatomic ternary phases ABC	990	113	1556	Accuracy: 96.9%	[24]
	SVR	ABO_3_ perovskites	15	6	128	RMSE: 1.08	[25]
	LR	Solid lithium-ion conductor materials	20	5	40	–	[26]
Hybrid	CA and GB	High-entropy alloys	54	3	162	–	[28]
	PCC and SC and LASSO	Chalcogenides	23 454	85	119	*R* ^2^: 0.9408	[29]
	PCC and LASSO	Functionalized MXene	47	8	70	RMSE: 0.14 eV	[30]
	Wrapper and PSO	Coal	27 620	114	840	*R* ^2^: 0.99	[31]

‘–’ indicates that this term is not mentioned. FCC, face-centered cubic materials; HCP, hexagonal close-packed materials; RMSE, root mean square error between the predicted value and actual value; AV, average of the actual value; RMSE/AV, a ratio that shows the overall deviation degree of the predicted sample; AUC, area under the receiver operating characteristic curve (ROC), a metric for binary classification.

Filter methods select the best features from a rank of the whole set of features by evaluating their intrinsic characteristics. Im *et al.* [10] employed the Pearson correlation coefficient (PCC) to select and confirm the 20 most important features for the bandgap. This enabled the root mean square error (RMSE) of the gradient-boosted regression tree to reach as low as 0.322. Note that Deng [11] employed PCC and the Spearman correlation coefficient (SCC) to explore the correlation between candidate descriptors and target property, whose results show that the valence state (descriptor) and lattice constants (target property) in cubic perovskite oxides had no correlation and thus the former were eliminated from the model. Agrawal *et al.* [12] leveraged an information-entropy-based (IE) metric to measure the features with respect to the target variable and selected the proper features for modeling. The results show that, with the favor of it, the multivariate polynomial regression obtained the best prediction performance (*R*^2^ = 0.98). Shin *et al.* [13] employed the maximal information coefficient (MIC) to calculate the correlations between descriptors, then 21 descriptors were selected from 466 candidates. The results show that better ML performances can be obtained based on MIC than those based on PCC, whose accuracy reaches >90%.

Compared with filter methods, embedded methods perform FS during the process of model construction [14,15] and greatly simplify the process of FS, which is limited to specific ML models (decision-tree-based and linear models). Qi *et al.* [16] employed the L1 norm method for FS to avoid overfitting in the regression tree model for the prediction of the carbon fiber mechanical properties. Zeng *et al.* [17] utilized gradient boosting regression to quantify the feature importance, then 14 features were chosen out of 21 candidate features, whose regression model achieved high accuracy (*R*^2^ = 0.90). Stanev *et al.* [18] employed random forest (RF) to quantify the feature importance, whose results show that RF using only the five most informative features can still achieve almost 90% accuracy. O’Connor *et al.* [19] utilized least absolute shrinkage and selection operator (LASSO) regression to select 75 descriptors for the accurate prediction of binding energy from 333 932 candidate features, achieving an RMSE of 0.4096. Mangal *et al.* [20] employed several FS methods to determine which microstructural characteristics can cause stress to build up in certain grains during uniaxial tensile deformation. The results show that the FeaLect algorithm, an improvement over the LASSO algorithm, was the most suitable and enabled feature rankings for physical interpretations.

Wrapper methods are model-dependent as well and are generally used in combination with a specific ML model and a meta-heuristic algorithm to identify the best feature subset without sacrificing prediction accuracy [21]. This method can be used with any ML model and can find the feature subset that enables the ML model to achieve optimal or near-optimal prediction performance in a huge feature space through search techniques. Wu *et al.* [22] employed different ML models to investigate the best combinations of descriptors for face-centered cubic solute diffusion predictions. The results show that artificial neural networks (ANNs) gained the lowest RMSE (0.092), selecting 14 optimal descriptors from 111 candidate descriptors. Furmanchuk *et al.* [23] employed RF to select the proper number of descriptors from 452 candidate descriptors. The results show that the RMSE of RF in the context of the 187 selected descriptors (0.92) surpassed that of RF based on all the descriptors (0.81). Oliynyk *et al.* [24] utilized a cluster resolution FS to select the best combination of variables in conjunction with a support vector machine (SVM). The results show that only 113 features out of 990 were sufficient to gain optimal classification performance of the SVM (Accuracy = 96.9%). Xu *et al.* [25] utilized three ML models to explore the best subset of features from 15 candidate features, whose results show that support vector regression (SVR) was able to achieve the lowest RMSE (1.08) tested on the data set with six features. Sendek *et al.* [26] performed FS via logistic regression (LR) from 20 candidate features. As a result, a five-feature model with a minimal cross-validated misclassification rate was constructed to screen out 21 structures that showed promise as electrolytes from 12 831 candidate materials.

The hybrid methods have the advantages of both the filter and the wrapper methods [27]. The filter approach is first employed to identify the best relevant features of the data sets. Then, the wrapper method is employed to verify the previously identified relevant feature subsets by using a method that gives higher classification accuracy rates. Wen *et al.* [28] employed a hybrid method combining correlation analysis and a gradient boosting algorithm to remove 14 features from the original pool of 54 features. Then, physics-based models and several ML models were employed to perform FS so that three features were determined to build a new model for the accurate prediction of solid solution strengthening. Wang *et al.* [29] employed hybrid methods combining PCC, Spearman correlation (SC) and LASSO to select 85 descriptors from 23 454 candidates and developed a stacking regression model (GBDT) using 119 compounds that efficiently predicted the band gaps of diamond-type-structure chalcogenides. Rajan *et al.* [30] employed PCC to quantify the feature correlation, then LASSO was used to further reduce the feature quantity down to eight features, which yielded a Gaussian process regression (GPR) with the lowest RMSE of 0.14 eV. Yan *et al.* [31] proposed a hybrid FS approach, combined with wrapper and particle swarm optimization (PSO), to select an optimal subset to model Laser-induced breakdown spectroscopy data. The results show that this method could select the optimal feature subset (114) from the original 27 620 candidates.

#### FT

FT generates new features via mapping original features into a space with lower dimensions, i.e. dimensionality reduction. Dimensionality reduction originates from the latent semantic index model [32]. It aims to transform feature values into a certain pattern, change the spatial relations of original features and obtain new features by analysing the relations between the original ones. Examples of FT in materials science are presented in Table 2.

**Table 2.** Examples of feature transform.

Methods	Materials	Number of raw features	Number of governed features	Number of samples	Results	Reference
PCA	Battery	5	1	34	Accuracy: 99.7%	[34]
	Binary metallic alloys	114	9	55	RMSE: 50 meV	[35]
SISSO	Binary systems	6	2	299	Accuracy: ∼99%	[36]
	Perovskite oxides and halides	9	2	576	Accuracy: 91%	[37]
	Catalytic materials	18	8	211	RMSE: 0.18	[38]
	Inorganic crystalline solids	19	3	309	RMSD: 61 meV/atom	[39]
GPSR	Oxide perovskite catalysts	1808	1	∼8640	–	[40]
	MXene materials	25	16	25	–	[41]

‘–’ indicates that this item is not mentioned. RMSD, root mean square deviation.

Principal component analysis (PCA) [33] is an unsupervised linear dimensionality reduction method that employs covariance as a measure to remove noise and redundant features to the greatest extent. Sturlaugson *et al.* [34] transformed five initial features into one feature via PCA to simplify the Bayesian network (BN) model of diagnostics on lithium-ion batteries. The results show that the average accuracy of the BN model with PCA achieved ≥10% improvement over the model without PCA. Curtarolo *et al.* [35] employed PCA to transfer 114 features into nine dimensions, which enabled partial least squares regression to describe the energies with a RMSE of 50 meV.

Sure independence screening (SIS) based on correlation learning is effective for the dimensionality reduction of ultra-high-dimensional feature spaces, which scores each feature with a correlation magnitude and keeps only the top-ranked. The SIS and sparsifying operator method called SISSO autonomously finds the optimal *N*-dimensional descriptor through sparsifying operators after SIS. Ouyang *et al.* [36] utilized SISSO to construct a 2D descriptor from a six-dimension descriptor, which enabled SVM to classify metal and nonmetal materials with a training accuracy of ∼99.0%. Bartel *et al.* [37] used SISSO to obtain a tolerance factor descriptor to predict the stability of perovskites by using only the atomic oxidation states and ionic radius, achieving an overall accuracy of 91%. Andersen *et al.* [38] employed SISSO for descriptor identification and obtained 8 of the most proper features from 18 candidates. The results show that SISSO with eight descriptors achieved the lowest RMSE (0.18 eV). Bartel *et al.* [39] used SISSO to find a physical descriptor for the inorganic crystalline solids Gibbs energy, of which the simple descriptor based only on the atomic volume, reduced mass and temperature reached a root mean square deviation of 61 meV/atom.

In addition, generating new features by employing basic mathematical functions alongside the original features can be regarded as an FT approach as well, such as genetic programming-based symbolic regression (GPSR). Weng *et al.* [40] generated effective descriptors using GPSR, which identified the combination of the tolerance factor (*t*) and the octahedral factor (*μ*), *μ*/*t*, as a primary descriptor for predicting the oxygen evolution reaction activity of oxide perovskites quantitatively. He *et al.* [41] employed symbolic regression to select a new relevant descriptor for a fast and efficient method to perform the classification of MXene materials and design new descriptors.

### Sample quantity governance

There have been some efforts towards data accumulation to build large databases to capture results of high-throughput computation and high-throughput experiments. However, in many materials studies, especially of novel materials, the problem of insufficient data to construct reliable ML models is still often faced [42]. To this end, sample quantity governance is utilized. This type of method focuses on treating original samples (i.e. sample-oriented method) or modifying the learning algorithms that enable ML models to extract useful information from small samples (i.e. model-oriented method).

#### Sample-oriented method

*Data augmentation.* Data augmentation commonly refers to data expansion of the original small sample data set with the help of auxiliary data or information [43]. Typically, researchers add Gaussian or impulse noise into the samples to increase the diversity of the initial samples [44]. However, this can limit the diversity of the augmented samples with numerous samples without any practical value generated. With the development of DL, neural-network-based methods such as the variational auto-encoder (VAE) and GAN have been successfully applied to data augmentation and have achieved better performance in materials science, examples of which are summarized in Table 3. Thereunto, Song *et al.* [45] employed GAN to generate 2.65 million 2D material samples, then discovered 26 489 new potential 2D materials, among which 1 485 2D materials had an area under the curve (AUC) of >0.95. Dan *et al.* [46] employed GAN to generate novel hypothetical inorganic materials that are not recorded in existing databases to enable the inverse design of such materials. Ma *et al.* [47] utilized GAN to expand the original 47 images of polycrystalline iron to 136, of which the model performance on the data sets composed of generated data and 35% real data were comparable to those on all real data. This demonstrates the feasibility of combining generated data with real experimental data. As for VAE, Noh *et al.* [48] employed VAE to perform an inverse design of solid-state materials. The results show that ∼20 000 hypothetical solid-state materials are generated via VAE, leading to several completely new metastable V_x_O_y_ materials that may be synthesizable. Hoffmann *et al.* [49] utilized VAE trained on >120 000 3D positions of atoms in a molecule to generate new materials.

**Table 3.** Examples of data augmentation.

Methods	Fields	Number of features	Number of raw samples	Number of governed samples	Results	Reference
GAN	2D materials	8^*^85	291 840	2 650 264	AUC: 0.96	[45]
	Inorganic materials	8^*^85	251 368 (OQMD) 57 530 (MP) 25 323 (ICSD)	1 831 648 (OQMD) 1 969 633(MP) 1 983 231 (ICSD)	–	[46]
	Polycrystalline materials	400^*^400	47	136	MAP: 0.586	[47]
VAE	Inorganic materials	32^*^32^*^32	10 981 (MP)	∼20 000 (MP)	–	[48]
	3D molecules	30^*^30^*^30	46 744	–	–	[49]

‘–’ indicates that this item is not mentioned. AUC, area under the receiver operating characteristic curve (ROC), a metric for binary classification; MAP, mean average precision; OQMD, Open Quantum Materials Database; MP, Materials Project platform; ICSD, Inorganic Crystal Structure Database.

Although the data-driven methods achieved impressive results, they have limitations. For example, the VAE-based methods compress the input data via an encoder to extract the concept of data. Then, the decoder restores the input data according to the concept. In this process, the output data are slightly different from the initial data and can be regarded as new data. However, the augmented data may drop details during the process of encoding because the concept only contains the most representative eigenvectors. Moreover, the VAE-based method fails to learn the physical information from the materials data, which is also a common drawback of the neural-network-based method. GAN-based methods generate data from Gaussian noise via a generator, then the discriminator distinguishes the generated data and initial data. By this means, GAN can learn the complicated rules from the original data, then apply the learned rules to generate new samples with the target properties. Note that the obvious limitation of GAN is that only a Gaussian distribution can be leveraged to mimic the initial data. However, in reality, the materials data are complicated so the generation process may not be valid for a given material system. Moreover, differently from VAE-based methods that generate data according to the feature vector of the data, the data generation of GAN is from zero (noise) to one (valid data), which may result in low robustness.

*Active learning.* Active learning is a method that allows an efficient iterative search in the search space to identify candidate objects. In materials science, active learning employs pre-built ML models to sample candidate chemistry spaces iteratively and adaptively. It provides the most valuable candidate samples for costly calculations or experimental validation to accelerate the screening for novel high-performance materials. Therefore, for cases in which the data set is sample-scarce or difficult to generate, it can effectively overcome the problem of poor predictive power due to the limited number of samples, and thus effectively explore materials with the target conditional properties [50]. Examples of using active learning in materials science are shown in Table 4. Min *et al.* [51] employed active learning to screen inorganic ABO_3_ perovskite materials. The results show that through active learning with 30% of the whole data set (5218), >90% of the screening materials were satisfactory. Pruksawan *et al.* [52] employed active learning to select 15 new samples from 256 possible experimental conditions. The results show that, compared with the initial data set, the *R*^2^ of the gradient boosting model had an improvement of 25% (0.85). Doan *et al.* [53] employed active learning based on Bayesian optimization (BO) to perform the efficient identification of the desired homobenzylic ethers (HBEs), which screened 42 optimal HBE candidates from an unseen data set of 112 000 HBEs. Bassman *et al.* [50] used BO with a surrogate GPR from 500 candidate samples to find layered materials with desired properties.

**Table 4.** Examples of active learning.

Fields	Number of features	Number of candidate samples	Number of selected samples	Results	Reference
Inorganic perovskite	159	5218	79	–	[51]
Ni Ti-based shape memory alloy	4	256	15	*R* ^2^: 0.85	[52]
Homobenzylic ether	30	112 000	42	–	[53]
Layered material	12	500	–	–	[50]

‘–’ indicates that this item is not mentioned.

As active learning is performed based on the BO algorithm, its performance is determined by using surrogate models and utility functions of BO. Moreover, as per the ‘no free lunch theorem’ [54], there is no combination of surrogate models and utility functions that can be equally suitable for all materials research and thus additional optimization of the method is required for particular models. Each iteration of BO needs to update the probabilistic agent model. Therefore, updating the probabilistic model is computationally expensive and often cannot be employed for practical tasks requiring real-time operation when the problem dimension is high or there is a large amount of data. Note that in the case of a high-dimension search space, BO has low effective dimensionality, namely there are only a few dimensions that determine the objective function while the rest have little or no influence.

#### Model-oriented method

*Ensemble learning.* Ensemble learning [8] is an algorithm that combines base classifiers to achieve better prediction performance. The main idea is to learn by cascading multiple weak learners and combining these test results together according to a certain strategy, which can obtain a better pattern recognition effect than a single classifier. According to the types of generation methods of individual learners, ensemble learning can be divided into the serial ensemble (e.g. boosting [55]) and parallel ensemble [56]. The boosting algorithm is prone to overfitting in the case of training data with noise. The bagging algorithm reduces variance by averaging the results of multiple models, which can effectively alleviate the overfitting problem. Therefore, bagging is commonly employed in materials science. Bagging utilizes the bootstrap algorithm [57] to realize the operation of sampling. The bootstrap algorithm belongs to the put-back sampling method aiming to obtain the distribution of statistics and confidence intervals. RF is built based on the decision-tree-based learner for bagging integration and random attribute selection is introduced in its training process. This can effectively achieve dimensionality reduction of the features while obtaining higher accuracy.

Here, we summarize and analyse the research on ensemble learning in materials science (shown in Table 5). Thereunto, Okafor *et al.* [58] compared different ensemble learning approaches (RF and XGBoost) in the study of the transmission prediction of infrared radiation from hydroxyapatite samples. The results show that RF outperformed XGBoost, but its computational cost was higher. Farooq *et al.* [59] proposed the use of the ensemble learning approach to predict the strength of high-performance concrete prepared from waste materials. This study compared several ensemble learning methods and individual models, of which the results show that the utilization of bagging- and boosting-based algorithms can improve the responsiveness of individual models, and the RF- and bagging-based models have better robustness. Yang *et al.* [60] investigated the problem of screening the influencing factors of steel mechanical properties via RF, which gains a high accuracy of an average absolute percentage error of 2.52% and a RMSE of 21.65 MPa. Ji *et al.* [61] established a hot-rolled strip steel product defect prediction model based on the ensemble learning method (improved RF) to extract the key process parameters affecting the product quality. The results show that the prediction of defects in hot-rolled strip steel is significantly improved for the improved RF compared with a single RF method.

**Table 5.** Examples of ensemble learning.

Fields	Number of features	Number of samples	Results	Reference
Hydroxyapatite	2	900	*R* ^2^: 0.9983	[58]
High-performance concrete	9	1030	*R* ^2^: 0.92	[59]
Strip steel	22	3534	MAP: 0.0252	[60]
Strip steel	11	2278	AUC: 0.92	[61]

AUC, area under the receiver operating characteristic curve (ROC), a metric for binary classification; MAP, mean average precision.

*TL*. TL is the application of knowledge learned in solving one problem (source domain) to a different but related problem (target domain). This algorithm aims to enable the model to obtain a better learning effect in new tasks [62]. Note that the precondition for TL to succeed is that the source domain is relevant to the target domain. In this process, the knowledge (namely parameters of ML models and prior distribution of source data) is transferred into models. According to the transferred knowledge, models can learn the latent patterns and gain faster convergence, higher robustness and high accuracy from small new samples. Examples of TL are presented in Table 6. Gupta *et al.* [63] transferred the deep knowledge representation from the abundant streaming potential data (from OQMD) to efficiently guide the design of materials with limited experimental data (from JARVIS). The results show that the TL model had the lowest mean absolute error (MAE) (0.0708) for the prediction of formation energy, which reduced the MAE by 26% compared with the model trained from scratch (0.0964). Bäuml *et al.* [64] used TL to classify the tactile material. The results show that in 10-shot learning the accuracy of the deep model reached 90.3%, outperforming classification without TL by >40%. Chen *et al.* [65] utilized TL on YOLOv3, a DL model for multi-target detection, to eliminate the overfitting. The results show that TL models gained 95.3% accuracy, which was ∼5% higher than the model trained from scratch. Wang *et al.* [66] trained a DL model to accurately predict HSE06 band gaps. The results show than the prediction performance of the TL model (*R*^2^ = 0.98) outperformed that of the ordinary DL model (*R*^2^ = 0.89). Ma *et al.* [67] transferred knowledge from the source task to H_2_ adsorption at 100 bar and 130 K (one target task) via TL, which enabled the average predictive accuracy on the target tasks to be improved from 0.960 (direct training) to 0.991 (TL).

**Table 6.** Examples of transfer learning.

	Number	Number of samples	Number of samples		
Fields	of features	in source domain	in target domain	Results	Reference
Materials science	86	321 140 (OQMD)	28 171 (JARVIS)	MAE: 0.0708	[63]
Tactile material	1000^*^16	30 classes (100 samples per class)	6 classes (100 samples per class)	Accuracy: 90.3%	[64]
Non-ferrous metal scrap	416^*^416	COCO data set [100]	920 multi-target image samples	Accuracy: 95.3%	[65]
Semiconductor material	–	1439 PBE band gaps	64 HSE06 band gaps	*R* ^2^: 0.98	[66]
Gas adsorption material	5	13 506 MOFs of H_2_	1000 MOFs of CH_4_	Accuracy: 99.1%	[67]

‘–’ indicates that this item is not mentioned. OQMD, Open Quantum Materials Database; JARVIS, Joint Automated Repository for Various Integrated Simulations; PBE, Perdew–Burke–Ernzerhof; HSE06, Heyd–Scuseria–Ernzerhof; MOFs, metal−organic frameworks; MAE, mean absolute error.

In conclusion, the efforts above provide an accessible approach to governing the data quantity by reducing the number of features, augmenting the number of samples or leveraging specific learning mechanisms. However, the governance results are limited by the one-side pattern. Hence, how to synergistically govern the data quantity is a more important issue to be considered.

## A SYNERGISTIC DATA QUANTITY GOVERNANCE FLOW WITH INCORPORATION OF MATERIALS DOMAIN KNOWLEDGE

**Figure 2.**
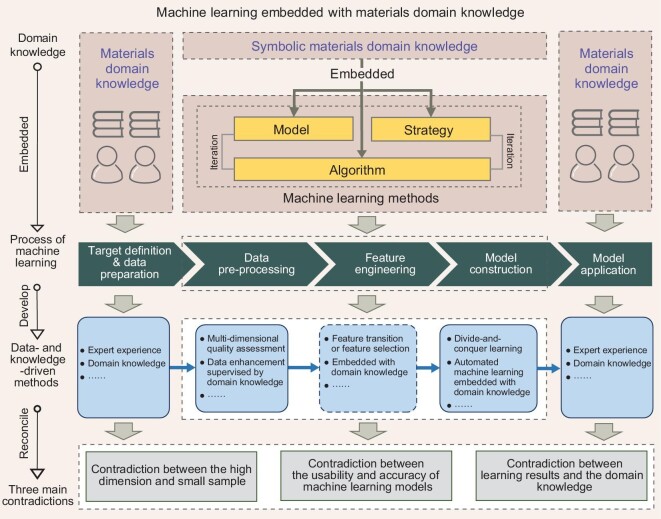
Framework of machine learning embedded with materials domain knowledge [68].

The application of data quantity governance methods to reconcile the contradiction between small data and high dimension has been a hot research topic in computer science [43] but so far has received little attention in materials science. In addition, the existing governance methods are purely data-driven, which often leads to contradictions between ML model prediction results and materials domain knowledge. To resolve these contradictions, the framework of Machine Learning Embedded with Materials Domain Knowledge [68] (Fig. 2) is proposed. In particular, this framework aims to perform symbolic representation of the materials domain knowledge and then embed it into the three key elements of ML (i.e. Model, Strategy and Algorithm). In this way, materials domain knowledge can be effectively embedded into the whole process of ML so that new ML models with high accuracy and explainability can be constructed and a high-coupled synergistic state can be realized in the paradigm of the ‘AI for Science (AI4Science)’ [69,70] in materials science. Based on this framework, we propose a general synergistic data quantity governance flow with the incorporation of materials domain knowledge, focusing on integrating materials domain knowledge into both feature and sample quantity governance, to ensure the interpretability, reliability and prediction accuracy of the resulting model.

### Domain-knowledge acquisition and representation

In conventional ML, materials domain knowledge mainly participates in data preprocessing or feature engineering. This method of participation is deeply intertwined with the learning process and thus cannot be employed as an independent source or through separated representations but is rather employed with adaption and as required. Nowadays, many studies focus on mining materials domain knowledge from materials science literature [71], while few studies focus on the integration of mined knowledge and the ML process.

**Figure 3.**
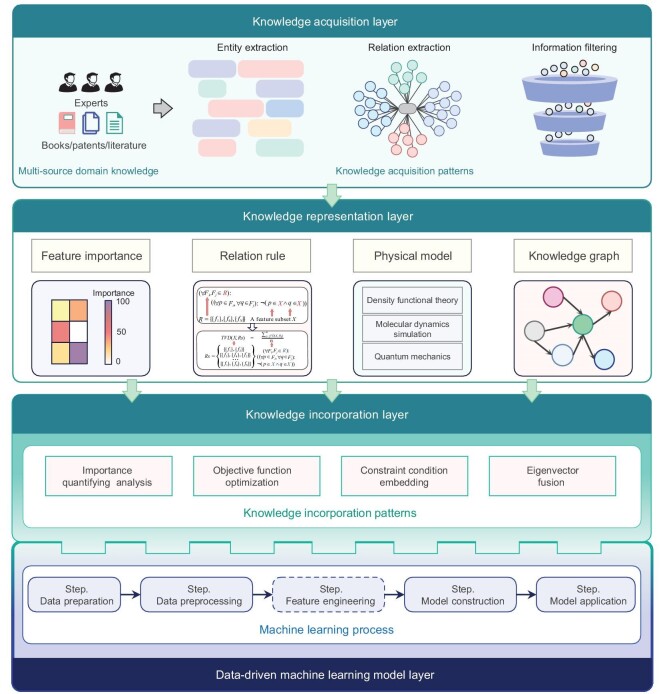
Schematic diagram of domain knowledge acquisition and representation. Feature engineering can be omitted in deep-learning schemes.

Here, we summarize the entire process of the approaches to domain-knowledge acquisition and representation (Fig. 3) that consists of knowledge acquisition, knowledge representation, knowledge incorporation and data-driven ML model layers. In the knowledge acquisition layer, multi-source domain knowledge can be extracted through an information filter [72] or approaches based on natural language-processing technologies such as entity extraction [73], relation extraction [74] and entity–relation extraction [75]. Then, the knowledge representation layer represents the extracted knowledge in the form of feature importance [76], relation rules [77], a physics model [78] or a knowledge graph [79]. Concretely, the feature importance analysis is quantified through domain experts, which can be combined with importance quantifying analysis technologies such as LASSO, RF, SHAP, etc. Relation rule can be employed to describe the correlation between descriptors and construct the conditions to eliminate redundant descriptors or assist the models in identifying the latent correlation relations. Based on the paradigm of ‘AI4Science’, performing the formulation representation of physical models and incorporating them into the learning algorithms of ML models can facilitate alternately optimizing the parameters of the neural network and the coefficients of the algebraic terms of the equation. Therefore, neural networks with high generalization can be trained to provide accurate derivative estimation, and finally ensure that the valuable information latent in the materials data can be mined. The knowledge graph can be extracted and regarded as rules [80]. This has been utilized in the neural-network-based models to encode the knowledge graph as an eigenvector and concatenate with original data [81]. By this means, ML models can be guided to mine the latent information in the original data. Note that one knowledge representation pattern can be modified to other patterns, determined by the demand for specific tasks [82]. To effectively embed mined domain knowledge into the process of ML models (i.e. data-driven ML model layer), the knowledge incorporation layer is constructed to provide various proper approaches. We here summarize these approaches into importance quantifying analysis [76], objective function optimization [83], constraint condition embedding [84] and eigenvector fusion [81,85]. Through the incorporation of various representations of domain knowledge into each step of data-driven ML, the domain knowledge can be effectively transformed and can participate the model training process so that the overall flow can achieve more reliable and accurate analysis results. More details of applications can be seen in Section S2 of the Supplementary data.

### Data quantity governance flow with incorporation of materials domain knowledge

As mentioned earlier, the sample size and the number of features jointly affect the performance of ML models. Therefore, we consider keeping the balance between sample size and feature space dimensionality as the data quantity governance objective. Concretely, on the one hand, high-dimensional materials data usually contain redundant features, but only pursuing the reduction of feature quantity may result in the loss of key features, which may aggravate the demand for more samples. Moreover, adding valid features into the data can be regarded as an effective approach to improving the performance of ML models [86], which requires abundant training samples. On the other hand, theoretically, the more samples provided, the more accurate the ML models will become. However, each sample, in fact, also adds noise or error information and thus may increase computation costs without providing insights. Therefore, data quantity governance should aim not at the sample and feature quantities separately but rather at the feature-to-sample ratio.

**Figure 4.**
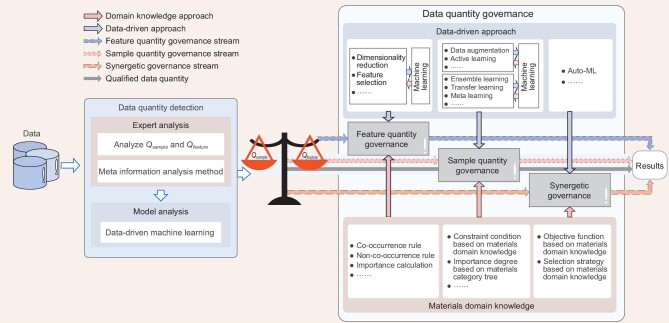
Data quantity governance flow embedded with materials domain knowledge.$\ {{{Q\!}}}_{{{\!Sample}}}$ represents the number of samples. $\ {{{Q\!}}}_{{{\!F\!eature}}}$ represents the number of features. Data quantity detection guides the relevant data quantity governance (feature quantity, sample quantity and synergistic governance). If the data quantity qualifies, the learning results of data-driven machine-learning models are directly outputted. Note that for DL models, ${{{Q\!}}}_{{{\!Sample}}}$ matters only; thus, data quantity governance merely focuses on the sample level.

As shown in Fig. 4, the data quantity governance flow with the incorporation of materials domain knowledge consists of data quantity detection and data quantity governance. The former aims to assess whether the data sets need to be governed from the perspectives of domain-knowledge and data-driven aspects. The latter performs targeted governance according to the detection results and is divided into feature quantity governance, sample quantity governance and synergistic governance.

#### Data quantity detection

To circumvent redundant operation, the data quantity detection module is constructed to effectively confirm whether the data need quantity governance. Note that the relevant materials domain knowledge that a researcher possesses affects whether the data quantity requires governance or not. Expert analysis is first performed based on materials domain knowledge to roughly estimate the characteristics of the data. Meta-information (metadata) not only contains basic information (e.g. the number of features, the number of samples and rules), but also reflects the statistical characteristics. Therefore, the stage of meta-information analysis here is performed, namely experts prejudge the metadata of data sets to estimate whether the number of samples can support the accurate analysis of ML models according to the number of features. Then, model analysis is performed, as shown in Supplementary Fig. S2. At that stage, if the number of samples is sufficient to drive DL models, then DL models can be regarded as detection tools directly, otherwise data-driven methods that are suitable for the analysis of high-dimension and small samples (e.g. SVR) are adopted. Depending on the analysis results, a suitable governance scheme is selected. Specifically, if the researchers are not familiar with the target tasks, it is correct to select the synergistic governance scheme to circumvent manual intervention.

#### Feature quantity governance with incorporation of materials domain knowledge

This module allows data-driven models to verify the effectiveness of feature quantity governance, and the governance methods can be further adjusted according to the analysis result of the data-driven models. Incorporating material domain knowledge into the process of feature quantity governance can help models to eliminate redundant features and construct more simple but efficient ML models. As the FT methods focus on feature space transformation, it is hard to embed existing symbolic domain knowledge into them. Herein, only FS methods are considered to illustrate the incorporation patterns of domain knowledge.

Liu *et al.* [76] proposed FS embedded with materials knowledge including data-driven multi-layer FS embedded with domain expert knowledge that combines the materials expert assessment of the importance of descriptors with data-driven measurement results, as shown in Supplementary Fig. S3. This method combines filter and wrapper methods to remove sparse, irrelevant and redundant features automatically. Then, the importance of descriptors is introduced into the FS process via domain expert knowledge to eliminate the risk of the deletion of key features. This has been shown to be effective in selecting descriptors with high prediction accuracy and consistently with expert knowledge.

In addition, materials domain knowledge can help to find correlation among descriptors. For example, Liu *et al.* [87] transferred the materials domain knowledge for the relationships between descriptors into non-co-occurrence rules (NCOR) and embedded NCOR into the process of FS. Then, a feature selection method is proposed to reduce correlations among features by embedding domain knowledge (NCOR-FS), as shown in Supplementary Fig. S4. The correlation between various factors affecting the ion transport performance of a solid electrolyte is transformed into NCOR and embedded into the objective function of the FS. In the ion activation energy prediction task of a NASICON-type electrolyte, the descriptor set with a lower internal correlation is successfully selected and a regression prediction model with better generalization performance and stability is constructed.

#### Sample quantity governance with incorporation of materials domain knowledge

Through sample quantity governance, the number of samples can be optimized, which effectively facilitates the application of data-driven methods. Its performance can be also verified through data-driven methods that are the same as the feature quantity governance module. Note that, in this module, either sample-oriented or model-oriented types are essentially driven by the ML model. Therefore, it can alleviate the contradiction of the small sample size and high dimensionality of the feature space (or model parameter space) to some extent without embedding materials domain knowledge. To further circumvent the model uncertainty, low interpretability as well as low robustness [88], sample quantity governance with incorporation of materials domain knowledge is proposed.

*Data augmentation*. Materials domain knowledge can be regarded as the constraint conditions for generative models to limit the distribution of generated samples with a reasonable value range. Concretely, for VAE-based methods, materials domain knowledge can be used in the form of formulas to be embedded into the training process. This facilitates the model catching the most important features vector, retaining the details of the data. Note that VAE and its variants are commonly utilized to ensure the explainability of input data via analysing the concept generated from the encoder [89]. Hence, materials domain knowledge can be also transformed into the physical or statistical formulas to preserve details of the input data. As for GAN-based methods, the Gaussian mixture model [90] can be employed to investigate the most representative Gaussian distribution of material data. In this way, GAN-based methods can generate valid samples according to the representative distribution. Then, materials domain knowledge is transformed into constraint conditions to eliminate the unreasonable samples. Besides, the original data can be first encoded by a neural network under the guidance of materials domain knowledge, whose form is the same as modified VAE-based methods. Besides, there are some approaches that can also provide insights for physical-informed data augmentation, e.g. the addition of perturbation physical information to original samples [91] and physical-based approaches (e.g. *ab initio* MD calculations) or simulation approaches for density functional theory (DFT) (e.g. projector-augmented wave) [92]. More details of applications can be seen in Section S3 of the Supplementary data.

*Ensemble learning.* The key idea of ensemble learning is combining several weak learners to obtain a comprehensive strong learner, which can circumvent the difficulty of directly constructing a strong learner. Based on this thought, Liu *et al.* [93] proposed the machine-learning-based method of ‘divide-and-conquer’, which can be regarded as a novel type of ensemble learning, to predict the creep rupture life of Ni-based single-crystal superalloys in the context of a small sample size and high dimension of feature space (shown in Supplementary Fig. S5). Concretely, to conquer the complicated distribution of the Ni-based single-crystal superalloys data set, this method leveraged the K-Means cluster method to divide the data set into different clusters according to their statistical characteristics. Then, appropriate models were automatically selected for each according to their individual characteristics. The results show that the divide-and-conquer method could be successful for a small sample size (266 samples) and high dimension of feature space (27 descriptors) and achieved a predictive accuracy of 91.76%, which surpassed that of ML models based on the original undivided data set. In the divide-and-conquer process, materials domain knowledge can guide cluster methods to group data sets according to chemical composition, processing conditions, etc. By this means, the data space with complicated influence mechanisms of materials can effectively divide the overall data set into data subspaces, which facilitates modeling for each of them. Finally, a simple ML model with high prediction performance and explainability will be obtained.

*Active learning.* BO constructs prior distribution through sampling parts of samples, thus the results depend on the sample quality and on how close the assumed sample distribution is to the true sample distribution. To improve the effectiveness of BO, it is possible to map the high-dimension search space into a lower one. For example, Wang *et al.* [94] employed BO to replace solving an ∼1 billion-dimensional problem with a large number of low-dimensional problems (the effective dimension is 2), which can be solved much faster. Li *et al.* [95] proposed a BO method for a high-dimension space that uses a projected-additive Gaussian process to solve high-dimensional problems. Moreover, material domain knowledge can be transformed into rules of distribution of materials data to improve its sampling procedures. For example, Xue *et al.* [96] embedded the knowledge of the morphotropic phase boundary providing a temperature-independent *d_33_* piezoelectric property for the compounds into the active learning loop. Then, the optimal solid solution composition was predicted and validated, which shows good temperature reliability. Yuan *et al.* [97] utilized active learning based on physical insights from the composition–temperature phase diagram to shrink the size of the virtual space from ∼9 million to 700 000. Note that there has been interest in the use of text mining and natural language-processing techniques to build data sets from the materials science literature for guiding materials synthesis and design [98]. Such advances can have key implications for the adaptive learning approach, especially in rapidly constructing training data sets for building surrogate models. Hence, integrating text mining for training set construction and active learning can have an impact in the accelerated search for novel materials.

*TL*. For incorporating materials domain knowledge into TL, we believe that calculating meta-information of the target domain and various candidate source domains ensures their relevance. All candidate source domains can be constructed as a tree based on materials domain knowledge. This enables each source candidate domain to get its scores from materials experts according to the need for the target source. By this means, the candidate source domain can be comprehensively analysed from the perspective of domain knowledge and statistics. As for the network structure, though DL can extract multi-scale and deeper features from materials data, the inexplicability of the model and difficulties in learning physical and geometric information are inevitable. Hence, the incorporation of materials domain knowledge can not only guide models to learn the latent pattern of materials data rapidly (i.e. fast convergence speed during model training), but also help materials experts to comprehend the learning process of the model.

#### Synergistic governance with incorporation of materials domain knowledge

Synergistic governance aims to balance the sample and feature quantity (or model parameter quantity) automatically. Automated machine learning (Auto-ML) [99] can meet this demand in that it can perform the steps of data processing, feature engineering and model construction automatically according to the specific tasks of materials design and discovery. That is, the procedure of synergistic governance is consistent with that of Auto-ML because sample quantity governance can be regarded as data processing. Meanwhile, feature quantity governance can be set as the step of feature engineering. Moreover, Auto-ML can reduce human intervention in the steps of model selection, optimization and implementation. According to the ‘no free lunch’ theorem [54], it is impossible for a single algorithm to be universally superior to any other algorithm. This implies that ML algorithm selection and hyperparameter setting differ in data sets with different characteristics (e.g. data size and distribution). Therefore, Auto-ML can help researchers who have low familiarity with the downstream tasks to gain the intended results. However, the conventional Auto-ML is purely data-driven, and knowledge incorporation can substantially improve the accuracy and explainability of the constructed models via synergistic governance. The knowledge incorporation pattern for synergistic governance can be seen in Supplementary Fig. S6 and the details of knowledge incorporation can be seen in Section 3.1.

Although the Auto-ML method based on meta-learning can automatically select the ML algorithm with ideal prediction performance by learning the historical modeling experience and greatly reduce the combined algorithm selection and hyperparameter optimization (CASH) time and calculation cost, the tedious training process of Auto-ML can hinder wide employment in materials science. To this end, a feasible Auto-ML scheme with materials domain knowledge is proposed here, to accelerate this process and improve its accuracy. Concretely, a new meta-feature is constructed to enhance the similarity measurement between data sets. The candidate ML algorithms suitable for materials property prediction are selected by investigating materials literature related to ML. As for limited materials data sets and the lack of domain knowledge, a collaborative learning mechanism with materials domain knowledge is employed to integrate ML public data sets and materials data sets for meta-learning. This alleviates the overfitting problem and improves the interpretability and reliability of ML models.

## CONCLUSIONS AND OUTLOOK

As ML models are widely employed in the field of materials science, the contradiction of a high dimensionality of feature space and a small sample size is increasingly encountered. The misbalance in data quantity (i.e. the number of samples vs. the number of features or model parameters) limits the performance of ML models both in prediction accuracy and the ability to mine latent patterns in the materials data. Here, we reviewed the efforts for data quantity governance in materials science such as feature quantity reduction, sample quantity augmentation and specific ML approaches. Then, a synergistic data quantity governance flow with incorporation of materials domain knowledge is proposed and corresponding approaches to knowledge representation and incorporation for ML are summarized. Benefitting from the incorporated materials domain knowledge, this flow can construct a high-quality data foundation to facilitate ML modeling. As for the development of a general generative artificial intelligence model (e.g. ChatGPT), it is promising that researchers can more effectively embed various pieces of domain knowledge into a sample generation process and materials samples can effectively be generated following their instructions, which can facilitate the development of AI4Science in materials science. Moreover, further development of ensemble tools for data quantity governance with the incorporation of materials domain knowledge (e.g. the flow or framework of data quality governance) can expedite progress in materials science. Through systematically governing the data quantity, researchers can conduct reliable and reproducible data analysis in an orderly manner, and then monitor the materials data quality and construct high-quality samples and high-accuracy ML models.

## Supplementary Material

nwad125_Supplemental_File
