# Supplementary material for: Data quantity governance for machine learning in materials science
Source: Natl Sci Rev. 2023 May 1;10(7):nwad125. doi: 10.1093/nsr/nwad125 (PMC10265966; doi:10.1093/nsr/nwad125)
Supplement: nwad125_Supplemental_File [file nwad125_supplemental_file.pdf]

Supplementary data for

data quantity governance for machine learning in materials science

Yue Liu[1,4], Zhengwei Yang[1], Xinxin Zou[1], Shuchang Ma[1], Dahui Liu[1], Maxim Avdeev[5,6] and Siqi Shi[2,3,*]

[1]*School of Computer Engineering and Science, Shanghai University, Shanghai 200444, China;*

[2]*State Key Laboratory of Advanced Special Steel, School of Materials Science and Engineering, Shanghai University, Shanghai 200444, China;*

[3]*Materials Genome Institute, Shanghai University, Shanghai 200444, China;*

[4]*Shanghai Engineering Research Center of Intelligent Computing System, Shanghai 200444, China;*

[5]*Australian Nuclear Science and Technology Organisation, Sydney 2232, Australia;*

[6]*School of Chemistry, The University of Sydney, Sydney 2006, Australia*

[*]**Corresponding author.** E-mail: sqshi@shu.edu.cn

S1. Supplementary Figures

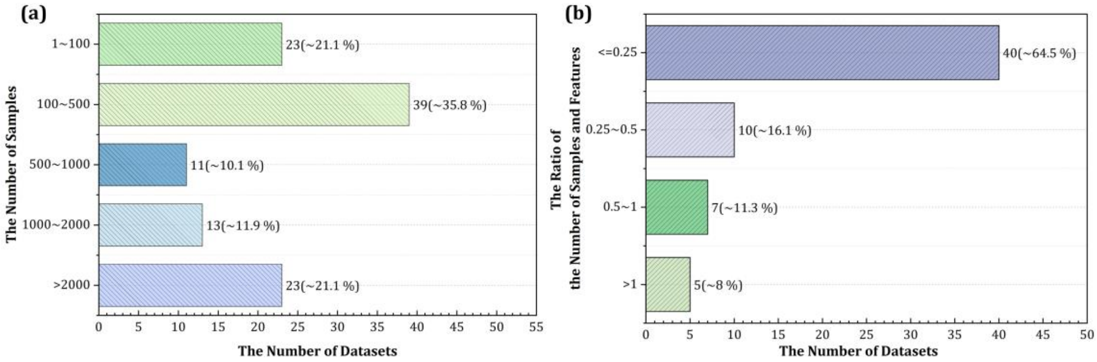

Figure S1. The Number of Samples and Features in 107 Papers in Materials Machine Learning. (a) The dataset size distribution. (b) The ratio of dataset size to the number of features

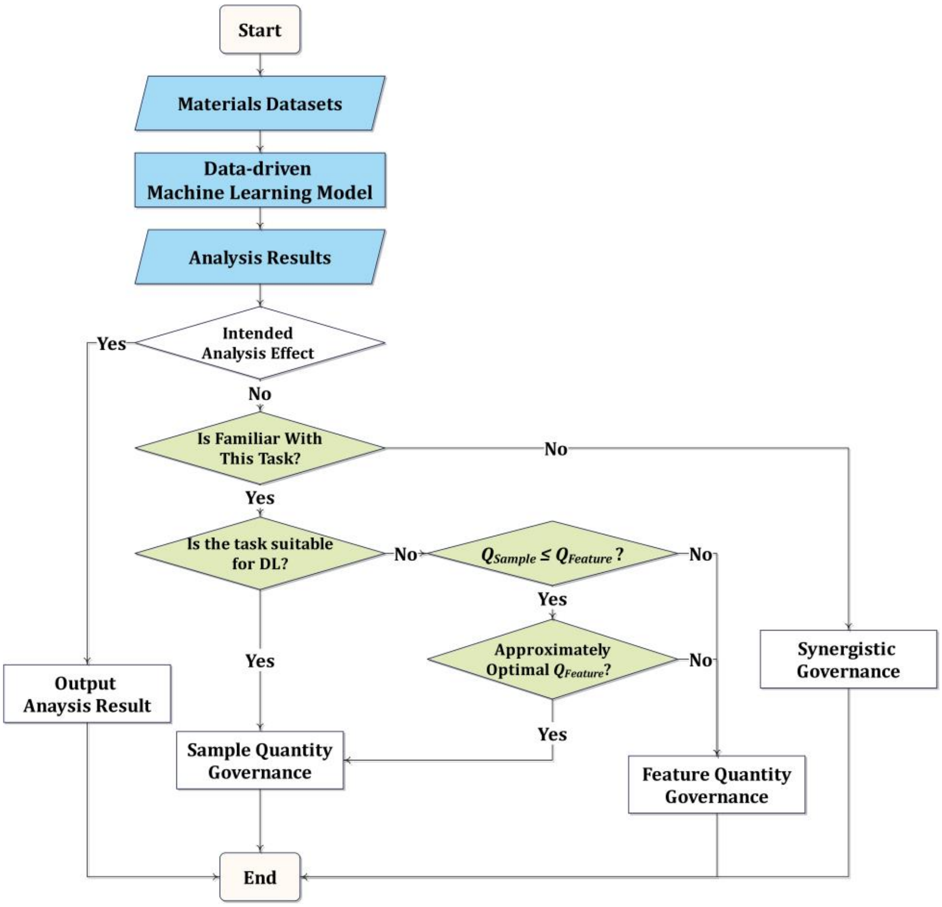

Figure S2. The Flow of Model Analysis Stage. The green background represents this step requires domain knowledge; The blue background represents this step is data relevant. $\boldsymbol{Q_{Sample}}$ represents the sample quantity; $\boldsymbol{Q_{Feature}}$ represents the feature quantity.

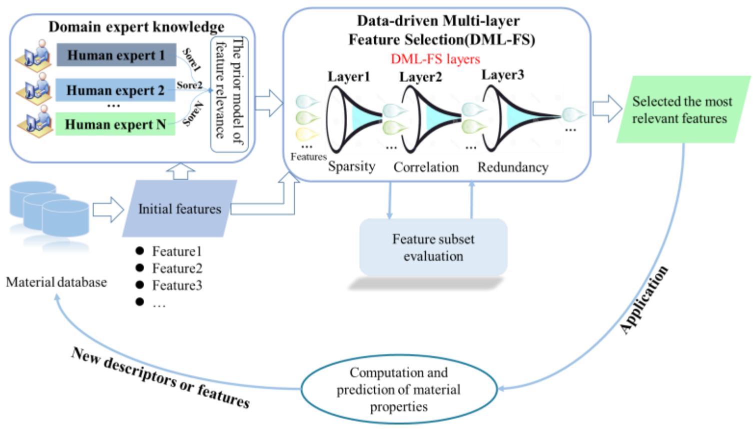

Figure S3. Data-driven Multi-layer Feature Selection Method Incorporating Domain Expert Knowledge [1].

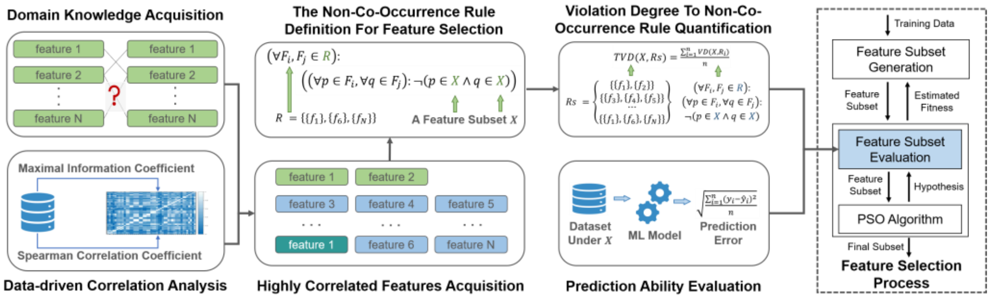

Figure S4. Flow diagram of NCOR-FS method. R and Rs represent a NCOR and the set of all NCORs, respectively. $f_1, \cdots, f_n$ represent features constructed by materials experts [2].

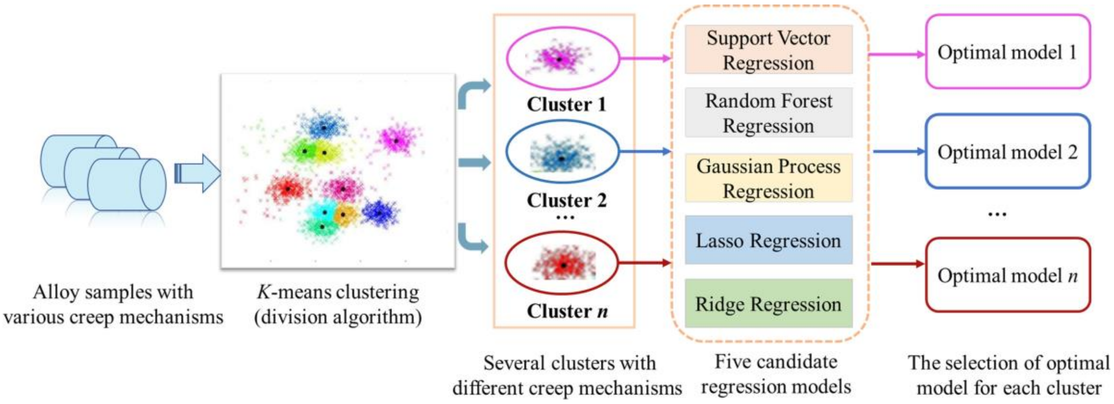

Figure S5. The procedure of divide-and-conquer self-adaptive (DCSA) learning method for modeling the creep rupture life [3].

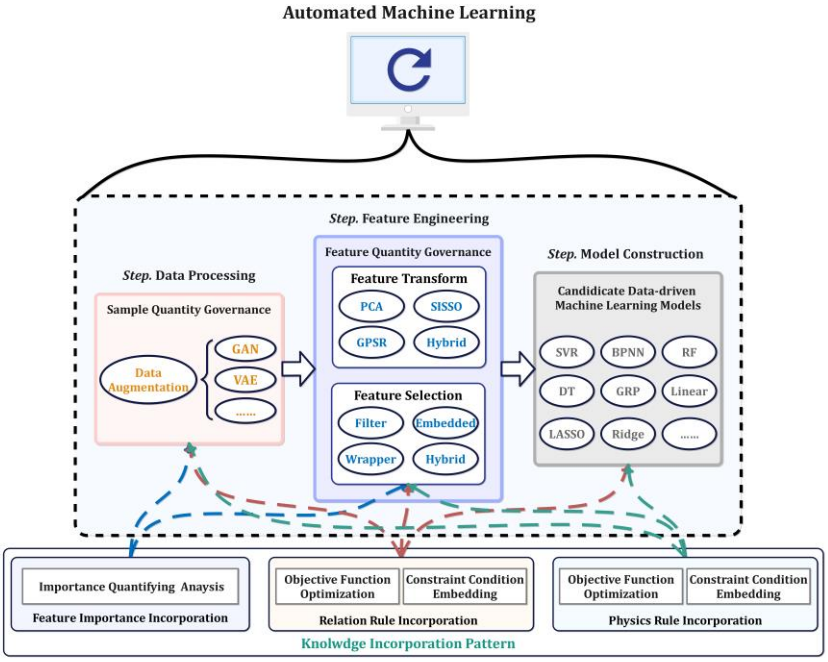

Figure S6. Synergistic Governance with Incorporation of Materials Domain Knowledge. The dummy line represents this incorporation pattern has not been used in this step.

S2. Examples of Knowledge Acquirement and Representation

This section details examples of knowledge acquirement and representation, which aims to facilitate the readers from broad and different background to comprehend the content of Section 3.1.

S2.1 Knowledge Acquirement

Ceder et al. [4] proposed an unsupervised approach to efficiently encode materials science knowledge present in the published literature as information-dense word embeddings. The results show that this method can recommend materials for functional applications several years before their discovery, suggesting that latent knowledge regarding future discoveries is to a large extent embedded in past publications, which points a novel path to extracting materials knowledge and relationships from scientific literature.

On this basis, Weston et al. [5] achieved automatic extraction of large-scale inorganic material information and solid-state synthesis information by manually annotating a large amount of supervised data and then training a deep learning NER model (BiLSTM-CRF), shown in **Figure S7**. The results show that their proposed model can effectively extract summary-level information from materials science documents, including inorganic material mentions, sample descriptors, phase labels, material properties and applications, as well as any synthesis and characterization methods used, which achieves 87% of identification accuracy.

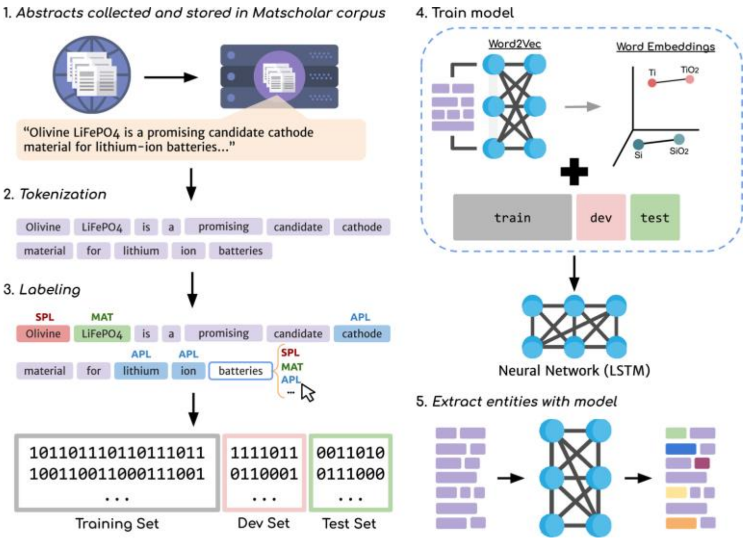

Figure S7. Workflow for named entity recognition [5].

Liu et al. [6] proposed an automatic descriptors recognizer based on natural language processing to mine latent descriptors (**Figure S8**), which can realize data augmentation with embedded domain knowledge from text data and filter task-related descriptors from coarse-grained to fine-grained. The results show that the proposed model can fully capture the contextual semantic features of the material text, classify words or phrases, and then use them for automatic recognition of descriptors. Finally, using the filtered descriptors, two datasets are constructed as activation energy predictions for ML models. These models have achieved good prediction results, demonstrating the effectiveness of automatic descriptor recognizers.

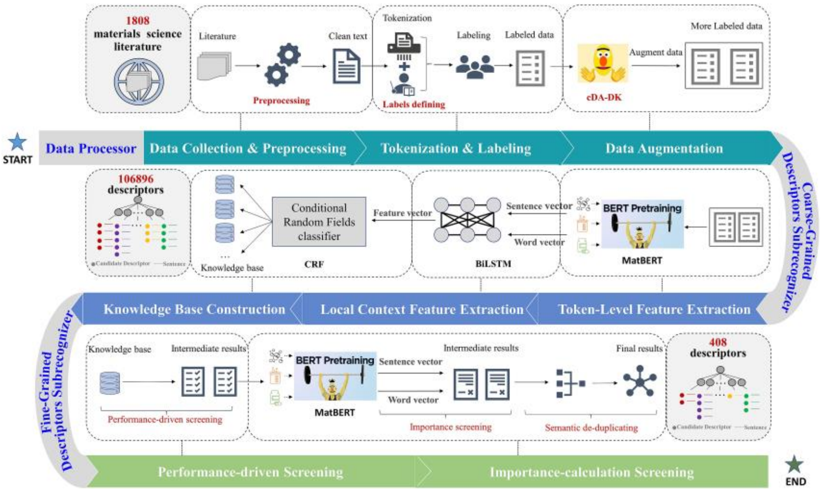

Figure S8. The overall pipeline of automatic descriptors recognizer [6].

S2.2 Knowledge Representation

Zhang et al. [7] proposed OntoProtein (**Figure S9**), a framework that integrates external knowledge graphs into protein pre-training, and proposed novel contrastive learning with knowledge-aware negative sampling to jointly optimize the knowledge graph and protein embedding during pre-training. The results demonstrate that efficient knowledge injection helps understand and uncover the grammar of life. Meanwhile, the proposed model is compatible with the model parameters of lots of pre-trained protein language models, which means that users can

directly adopt the available pre-trained parameters on OntoProtein without modifying the architecture.

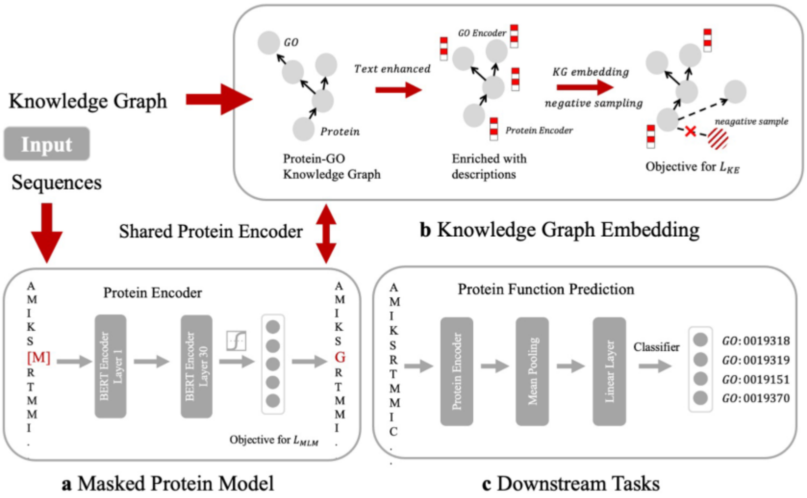

Figure S9. Overview of our proposed OntoProtein [7].

To discover governing partial differential equations from scarce and noisy data for nonlinear spatiotemporal systems, Chen et al [8] proposed a novel approach, physics-informed neural network with sparse regression (PINN-SR), shown in **Figure S10**, where objective function is comprised of data loss for descripting the difference between the measurement data and the corresponding DNN-approximated solution, physics loss for the description of the physical law, and regularization for accelerating the convergence. The results show that the proposed model can accurately discovering the exact form of the governing equation(s), even in an information-poor space where the multi-dimensional measurements are scarce and noisy.

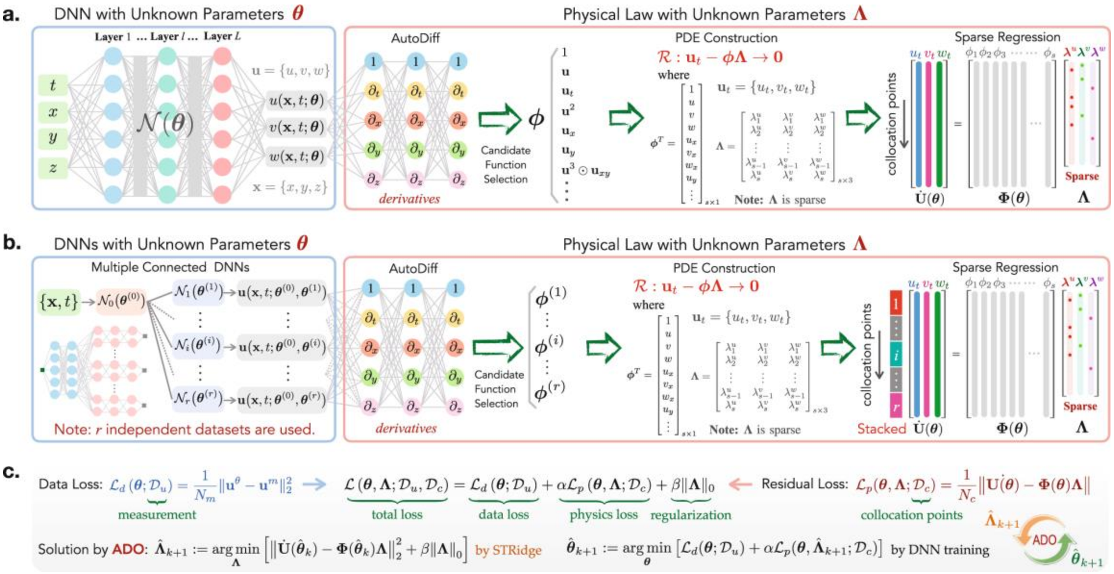

Figure S10. The framework of PINN-SR for data-driven discovery of PDE(s) [8].

To depict the heat energy fluxes and the lake thermal energy, Jia et al. [9] proposed PGRNN, which is embedded constraint condition as knowledge-based loss terms into the learning objective function. Moreover, generic general lake model (GLM) is employed to generate physical simulation data to pretrain the proposed model, aiming to leverage physical knowledge to help

inform the initialization of the weights, thus accelerating model training. The results show that the PGRNN can effectively model spatial and temporal physical processes while incorporating energy conservation.

Deng et al. [10] proposed knowledge-driven temporal convolutional network (KDTCN) for accurate stock trend prediction and explanation. Concretely, they extracted structured events from financial news and utilizes external knowledge from knowledge graph to obtain event embeddings, thus combining event embeddings and price values together to forecast stock trend. The results show that the proposed model not only can more accurately forecast stock trend with abrupt changes than present deep models but make explanation on prediction results with abrupt changes.

S3. Examples of Data Quantity Governance with Incorporation of Materials Domain Knowledge

This section details examples of knowledge acquirement and representation, which aims to facilitate the readers from broad and different background to comprehend the content of Section 3.2.

Gómez-Bombarelli et al. [11] applied VAE to the design of drug-like molecules, of which framework can be seen in **Figure S11**. By using an encoder network to convert a discrete molecular representation into a continuous vector in the latent space, and then performing simple operations on the latent continuous vector, such as perturbing known chemical structures, or interpolating between molecules. Then the modified vector can be converted back into a new discrete molecular representation through a decoder. Finally, a predictor module is used to predict the chemical properties of the new molecular representation, thus effectively searching for candidate materials with higher target performance.

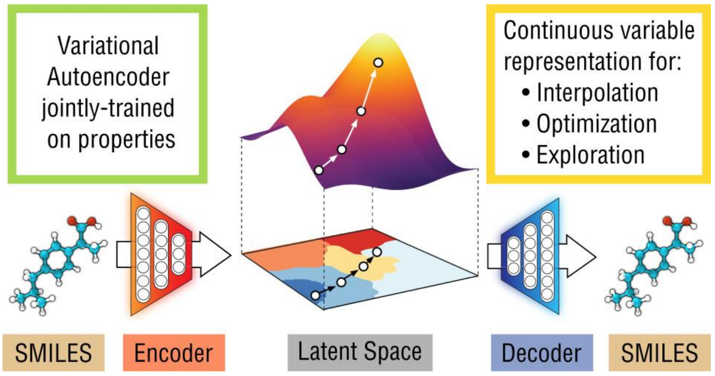

Figure S11. A diagram of VAE used for molecular design, including the joint property prediction model [11].

To improve the machine learning of this effective PES by better sampling the configuration space, Gibson et al. [12] add a perturbed structure for every relaxed structure and map it to the same energy as the relaxed structure, thus representing an additional point for a given basin of attraction of the energy landscape (shown in **Figure S12**). The results show that the prediction MAE of the CGCNN and CGCNN-HD were reduced from 251 meV/atom and 172 meV/atom to 86 meV/atom and 82 meV/atom, respectively, compared to training on only relaxed structures, which shows the surprising effectiveness of a relatively simple method of augmentations that outperformed the

current state of the art in formation energy prediction of unrelaxed structures.

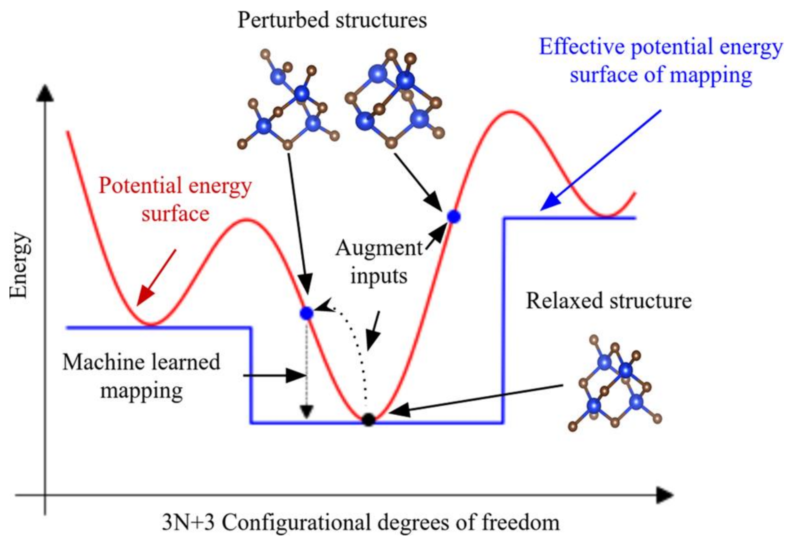

Figure S12. Data augmentation for learning the potential energy surface (PES) [12]. The red line denotes a 2D representation of the continuous PES of materials. The blue line illustrates the effective PES, which describes the energy of a relaxed structure for a given unrelaxed input structure. The black circle means the relaxed structures contained in the dataset, and the blue circles symbolize artificially generated structures for the data augmentation.

Moreover, to drive the accurate prediction of deep learning models, Li et al [13] generate plenty of monolayer graphene and monolayer MoS2 supercells by ab initio MD calculations and the Simulations are performed with the projector-augmented wave [14] pseudopotentials and the GGA parameterized by Perdew, Berke and Ernzerhof (PBE) [15].

Reference:

[1] Liu Y, Wu J and Avdeev M *et al*. Multi-layer feature selection incorporating weighted score-based expert knowledge toward modeling materials with targeted properties. *Adv Theor Simul* 2020; **3**: 1900215.

[2] Liu Y, Zou X and Ma S *et al.* Feature Selection Method Reducing Correlations among Features by Embedding Domain Knowledge. *Acta Mater* 2022; **238**: 118195.

[3] Liu Y, Wu J, Wang Z *et al.* Predicting creep rupture life of Ni-based single crystal superalloys using divide-and-conquer approach based machine learning. *Acta Mater* 2020; **195**: 454-67.

[4] Tshitoyan V, Dagdelen J and Weston L *et al.* Unsupervised word embeddings capture latent knowledge from materials science literature. *Nature* 2019; **571**: 95-8.

[5] Weston L, Tshitoyan V and Dagdelen J *et al*. Named Entity Recognition and Normalization Applied to Large-Scale Information Extraction from the Materials Science Literature. *J Chem Inf Model* 2019; **59**: 3692-702.

[6] Liu Y, Ge X, Yang Z, *et al.* An automatic descriptors recognizer customized for materials science literature. *J Power Sources* 2022: **545**: 231946.

[7] Zhang N, Bi Z and Liang X, *et al.* Ontoprotein: Protein pretraining with gene ontology embedding. arXiv:2201.11147.

[8] Chen Z, Liu Y and Sun H. Physics-informed learning of governing equations from scarce data. *Nat Commun* 2021; **12**: 6136.

[9] Jia X, Willard J and Karpatne A *et al.* Physics-guided machine learning for scientific discovery: An application in simulating lake temperature profiles. *ACM/IMS Trans on Data Sci*; **2**:1-26.

[10] Deng S, Zhang N, Zhang W, et al. Knowledge-driven stock trend prediction and explanation via temporal convolutional network. In: *Companion Proceedings of The 2019 World Wide Web Conference, San Francisco, USA*. New York: IEEE Press, 2019, 678-685.

[11] Gomez-Bombarelli R, Wei J and Duvenaud D *et al*. Automatic Chemical Design Using a Data-Driven Continuous Representation of Molecules. *ACS Cent Sci* 2018; **4**: 268-76. https://pubs.acs.org/doi/10.1021/acscentsci.7b00572.

[12] Gibson J, Hire A, and Hennig R G. Data-augmentation for graph neural network learning of the relaxed energies of unrelaxed structures. *npj Comput Mater* 2022; **8**: 211.

[13] Li H, Wang Z and ZOU N *et al.* Deep-learning density functional theory Hamiltonian for efficient ab initio electronic-structure calculation. *Nat Comput Sci* 2022; **2**: 367-77.

[14] Kresse G, Joubert D. From Ultrasoft Pseudopotentials to the Projector Augmented-Wave Method. *Phys Rev B* 1999; **59**: 1758.

[15] Perdew J P, Burke K and Ernzerhof M. Generalized Gradient Approximation Made Simple. *Physical Rev Lett* 1996; **77**: 3865.
